# Implementation of routine outcome measurement in child and adolescent mental health services in the United Kingdom: a critical perspective

**DOI:** 10.1007/s00787-013-0454-2

**Published:** 2013-07-30

**Authors:** C. L. Hall, M. Moldavsky, J. Taylor, K. Sayal, M. Marriott, M. J. Batty, S. Pass, C. Hollis

**Affiliations:** 1 CLAHRC, University of Nottingham, Nottingham, UK; 2 Nottinghamshire Healthcare NHS Trust, CLAHRC, University of Nottingham, Thorneywood, Nottingham, UK; 3 Division of Psychiatry, University of Nottingham, Nottingham, UK; 4 Specialist Community CAMHS, Nottinghamshire Healthcare NHS Trust, Nottingham, UK; 5 University Hospitals of Leicester NHS Trust, Leicester, UK; 6 Institute of Mental Health, Innovation Park, University of Nottingham, Nottingham, NG7 2TU UK

**Keywords:** Child and adolescent psychiatry, Routine outcome measurement, Implementation, SDQ, HoNOSCA, C-Gas

The aim of this commentary is to provide an overview of clinical outcome measures that are currently recommended for use in UK Child and Adolescent Mental Health Services (CAMHS), focusing on measures that are applicable across a wide range of conditions with established validity and reliability, or innovative in their design. We also provide an overview of the barriers and drivers to the use of Routine Outcome Measurement (ROM) in clinical practice.

For the purpose of this paper, we define ROM as the use of generic measures that assess the clinical outcomes or patient/carer satisfaction with service delivery. Outcome measures are usually completed at first contact (baseline) and after a fixed interval, often 6 months after the initial measure [1]. Symptomatic measures or measures of broader functioning that are completed only at one time point (e.g. at the end of intervention) do not provide a measure of within-individual change which is an essential feature of symptomatic or functional outcome measurement. The exception to this rule is measures of patient or caregiver satisfaction with the service which are typically obtained once at the end of treatment or discharge [2, 3].

The purpose and use of outcome measures may differ depending upon the end user of the data. ROM should enable clinicians to assess change over the course of treatment and help them draw comparisons between the perspectives of the clinician, child, their parent/carer and other informants such as teachers [4, 5]. Outcome measures provide service users with a way of seeing change in their condition and functioning over time and an opportunity to express their level of satisfaction with the care received [2]. At a service level, outcome data can help identify areas for development, evaluate whether services are meeting targets and influence the allocation of funding [5]. Anonymised outcome data collected at a service level may satisfy commissioners’ demand for greater service accountability through service user feedback and objective measurement of clinical effectiveness [2]. Regular, consistent outcome measurement should lead to improvements in practice and patient outcome, provided that results are carefully interpreted in the clinical and organisational context [6].

Fitzpatrick and colleagues [7] outline several criteria that outcome measures should meet. ROM should be based on measures with good psychometric properties, including established reliability, validity and sensitivity to change. Measures should be simple and quick to complete, cost-effective and easy to interpret. Furthermore, if outcome measures are to be used for benchmarking, they should be generic, relevant to the most frequent clinical diagnoses and applicable across a broad range of theoretical frameworks. Generic outcome measures do not cover factors specific to all disorders, but enable comparisons across disorders and services. Outcome measures data should be interpreted in the context of case mix and case complexity for each particular service.

The NHS National Service Framework for Children, Young People and Maternity Services [8] in England proposed that work conducted within CAMHS should be evaluated from the perspective of both clinicians and service users. In response to this, the CAMHS Outcome Research Consortium (CORC; [9]) was created to develop a common suite of measures and to provide leadership on CAMHS ROM and support to services with the collection and analysis of anonymised outcome data. CORC recommend a range of core outcome measures [10], including: the Strengths and Difficulties Questionnaire (SDQ) [11]; the Health of the Nation Outcome Scales for Children and Adolescents (HoNOSCA) [12] and the Children’s Global Assessment Scale (C-GAS) [13]. These measures were chosen for their established validity, reliability and applicability across a range of psychiatric problems in children and young people [10, 14] and open source access free of charge. CORC also recommend the use of two more recently developed measures whose psychometric properties are less well known: the Commission for Health Improvement-Experience of Service Questionnaire (CHI-ESQ) [14] as a measure of service user satisfaction, and the Goals Based Outcome (GBO) [15]. However, CORC do not advocate that ROM should be limited only to their selected measures [10].

Since 2011, CORC have been commissioned by the Department of Health to support the analysis of outcome measurements collated through the Children and Young People’s Improving Access to Psychological Therapies (CYP-IAPT; www.IAPT.nhs.uk). The CYP-IAPT aspires to improve services for service users by routinely assessing their opinion on the quality and experience of services. Alongside the SDQ, GBO and CHI-ESQ, CYP-IAPT recommend the use of brief scales such as the ORS (Outcome Rating Scale) [16] to measure functioning and the SRS (Session Rating Scale) [17] to assess client satisfaction on a session-by-session basis (http://www.iapt.nhs.uk/silo/files/cyp-iapt-outcomes-summary.pdf). Although there has been some research on the psychometric properties of the adult versions of these scales [18, 19], there is no research investigating the psychometric properties of these child versions.

Drawing on the work of Clark et al. [20] and Weiz et al. [21] CYP-IAPT specifically advocates the use of idiographic and standardised measures. In their commentary, Wolpert et al. [22] specifically make reference to the compromise of choosing measures which are sufficiently tailored to individual patient needs to be able to inform clinical practice whilst being broad enough to draw comparisons across cases and services. They also comment that the CYP-IAPT measures have been chosen with recognition of the need to reduce time burden for both the clinical staff and service users whilst balancing reliability and generalisability.

## Barriers to the implementation of ROM into clinical practice

The literature reveals common themes that are recognised barriers and facilitators to the implementation of ROM in mental health services internationally.

Studies have shown that treatment outcomes are measured in only 16–30 % of clinical cases [2, 3] in the UK, and only 37 % of psychologists in the US [23] reported measuring outcomes routinely; these findings suggest the presence of barriers to ROM in practice. Several studies have identified multiple barriers, including the increased time demands on clinicians and administrative staff [2, 3, 17, 22, 24]; a lack of clinician training on how to integrate ROM into clinical practice [3]; whether ROM is considered to produce clinically useful information [19, 23, 25, 26]; and a poor return rate of questionnaires completed by service users outside the clinic [27–29]. The frequent lack of timely feedback from outcome measures decreases their clinical usefulness and has a negative impact on clinicians’ and patients’ motivation to use them [2].

Additional barriers are related to limitations of the available outcome measures, for example, the fact that generic outcome measures do not typically assess self-harm behaviour and suicidal risk [30], or are not sensitive to symptom change in some clinical presentations [31].

Clinicians have expressed concerns about the time required to complete the questionnaires during the session [2, 32] and about outcome measures not being necessary [33] or relevant to their practice [23, 34]. Differences in psychologists’ willingness to use outcome measures according to their therapeutic approach (i.e. cognitive behavioural or insight-oriented) have been reported [23], and some clinicians may be reluctant to use a quantitative, systematic approach for data collection [35]. Johnston and Gowers [3] found that clinicians who did not regularly use quantitative clinical measurements were more likely to be sceptical about the value of the quantitative ‘medical’ approach. Additional concerns relate to **‘**labelling’ patients [30], confidentiality [23], and the risk of data being used by managers and commissioners to unfairly compare services that deal with different levels of case complexity [23, 24, 32]. Despite these reservations, most studies [2, 24, 36] report a range of clinicians’ views, with a substantial number of clinicians showing a positive attitude towards the implementation of ROM. Clinicians’ attitudes towards ROM have been shown to become more positive following attendance at a workshop and training focusing on their clinical value [37].

Studies have reported [34] that parents feel that ROM can add to the burden of form-filling already required of service users, even when language is not a barrier [38]; however, positive views about the opportunity to express their opinion have also been reported [2].

In summary, research shows that clinicians and service users have a range of views about ROM and identifies the need for further clinician training on the use of outcome measures, as well as a system to improve the provision of timely feedback from those measures to clinicians and patients/carers to support real-time clinical decision-making.

Future directions for development include the validation of session-by-session outcome measures [22] and the use of technology (for example, computer-based measures) to aid ROM implementation and reduce administrative burden. Research into the barriers to the integration of ROM in CAMHS in different countries will be of international interest and may provide insights into methods that support wider uptake of ROM and further evidence for their contribution to improved clinical effectiveness of child and adolescent mental healthcare.
